# Volunteering in the care of people with severe mental illness: a systematic review

**DOI:** 10.1186/1471-244X-12-226

**Published:** 2012-12-13

**Authors:** Claudia Hallett, Günter Klug, Christoph Lauber, Stefan Priebe

**Affiliations:** 1 Academic Unit for Social and Community Psychiatry, Barts & the London School of Medicine and Dentistry, Queen Mary University of London, Newham Centre for Mental Health, London E13 8SP, UK; 2 Psychosocial Services, Society of Mental Health Promotion, PSD Graz East, Hasnerplatz 4, Graz, A-8010, Austria; 3 Department of Psychiatry, University of Liverpool, 2nd Floor Block B, Waterhouse Building, 1-5 Brownlow Street, Liverpool, L69 3GL, UK

**Keywords:** Volunteering, Severe mental illness (SMI), Social support

## Abstract

**Background:**

Much of the literature to date concerning public attitudes towards people with severe mental illness (SMI) has focused on negative stereotypes and discriminatory behaviour. However, there also exists a tradition of volunteering with these people, implying a more positive attitude. Groups with positive attitudes and behaviours towards people with SMI have received relatively little attention in research. They merit further attention, as evidence on characteristics and experiences of volunteers may help to promote volunteering. The present paper aims to systematically review the literature reporting characteristics, motivations, experiences, and benefits of volunteers in the care of people with SMI.

**Methods:**

In November 2010, a systematic electronic search was carried out in BNI, CINAHL, Embase, Medline, PsycINFO, Cochrane Registers and Web of Science databases, using a combination of ‘volunteer’, ‘mental health’ and ‘outcome’ search terms. A secondary hand search was performed in relevant psychiatric journals, grey literature and references.

**Results:**

14 papers met the inclusion criteria for the review, with data on a total of 540 volunteers. The results suggest that volunteers are a mostly female, but otherwise heterogeneous group. Motivations for volunteering are a combination of what they can ‘give’ to others and what they can ‘get’ for themselves. Overall volunteers report positive experiences. The main benefit to persons with a psychiatric illness is the gaining of a companion, who is non-stigmatizing and proactive in increasing their social-community involvement.

**Conclusions:**

The evidence base for volunteers in care of people with SMI is small and inconsistent. However there are potential implications for both current and future volunteering programmes from the data. As the data suggests that there is no ‘typical’ volunteer, volunteering programmes should recruit individuals from a variety of backgrounds. The act of volunteering can not only benefit people with SMI, but also the volunteers. Further research may specify methods of recruiting, training, supervising and using volunteers to maximise the benefit for all involved.

## Background

Much of the literature to date concerning public attitudes towards people with a mental illness has focused on negative stereotypes and discriminatory behaviour [1-5]. There have been reports of landlords refusing to lease properties to people with a mental illness [6-8], and employees withholding job opportunities [6,7,9]. Such social distance behaviours reflect the mental health illiteracy that exists amongst the general public [10]. Public beliefs about the causes and presentations of mental illness are so often misinformed and misunderstood, that the challenge is to find ways of improving public knowledge in order to produce effective behaviour change [11-13].

Angermeyer & Dietrich (2006) conducted a review examining public attitudes towards persons with a psychiatric illness. Although their conclusions state there is still an observable tendency for people to distance themselves from individuals with a mental disorder, and that there is still a perception of people with a mental illness as ‘unpredictable and dangerous’, they allude to a substantial cohort of the public who hold ‘positive attitudes’ and demonstrate ‘pro-social behaviour’. Yet, groups with positive attitudes and behaviours towards people with mental illness have received relatively little attention in research.

One group in which positive attitudes are implicit are volunteers in mental health care [14,15]. Volunteering England, an independent charity committed to supporting volunteering defines ‘volunteering’ as ‘any activity that involves spending time, unpaid, doing something that aims to benefit the environment or someone (individuals or groups) other than, or in addition to, close relatives’ [16]. In 2010 it was estimated that 25% of the adult population in the United Kingdom (UK) volunteered formally at least once a month in the preceding 12 months [17]. About 3.4 million people have been estimated to volunteer in the UK Health Sector alone [18]. In the context of mental health care, volunteers are members of the public who intentionally seek out contact with and provide care to individuals with a mental illness. One type of one-to-one volunteering activity is ‘befriending.’ Befriending contact involves joint social and recreational activities, such as visiting sites of interest, sharing meals or playing sport. The relationship is typically initiated, supported and monitored by an agency that has defined one or more parties as likely to benefit. Ideally the relationship is non-judgemental, mutual, and purposeful, and there is commitment over time [19]. Exact figures on the numbers of volunteers in mental health care worldwide are difficult to obtain, but they are substantial as examples from the localities of the authors of this review demonstrate. In the Austrian region of Styria with a population of 1.2 million, one voluntary organisation alone has 298 volunteers who work directly with people with mental illnesses (Leitner P. Chief Executive of voluntary organisation ‘Pro Humanis’, personal communication). A Trust providing mental health services within the National Health Service in East London (population 750,000) recruited 250 new volunteers within their first seven months of operation (Lacey A. Volunteer Coordinator, East London NHS Foundation Trust, personal communication). Given the overall negative attitudes towards people with mental illness in the general public, the question arises as to what is distinct about mental health volunteers. They might hold pre-existing positive attitudes towards people with a mental illness or perhaps lack stigmatizing views all together.

There is also a practical interest in research evidence on volunteering. Although volunteers can still generate costs to services, e.g. for training and supervision, by definition they do not draw a salary and are a relatively inexpensive resource to deliver some aspects of care. Having said this, due care must be taken when involving volunteers in mental health services, so that they are not exploited or used as a means to ‘undercut on cost by substituting for pre-existing paid jobs or carrying out tasks that, by law, require clinical or professional training’ [20] as noted by a recent UK Department of Health report. Volunteers may provide people with a psychiatric illness with an experience that is distinct from and more ‘normal’ than their regular contacts with mental health professionals, and in this way help to facilitate their social inclusion.

This article presents an integration of available evidence on (i) the characteristics of volunteers in mental health care, (ii) their reasons for volunteering (iii) their experiences, and (iv) the benefit of volunteering schemes for people with a mental illness. These components were selected for study as we felt that they would be of most interest to volunteer organizations when thinking about recruiting and making best use volunteers.

## Methods

In November 2010, a systematic search of the literature was conducted using online databases, relevant psychiatric journals and grey literature.

For the electronic search, three lists of search terms were created:

a) ‘volunteer descriptors’, including: volunteer, lay helper, befriender, voluntary/informal caregiver, paraprofessional, nonprofessional, psychosocial support, intentional friendship, naturalistic contact, community support, and citizen/civic participation;

b) ‘mental health descriptors’, including: severe mental illness, schizophrenia, psychosis, psychotic symptoms, mental disorder, mental health charity, mental health project, mental health programme, psychiatric scheme, and psychiatric organisation;

c) ‘outcome descriptors’, including: motivation, reason, opinion, attitude, experience, reward, and challenge.

The full lists of terms can be obtained from the authors.

### Search strategy and selection criteria

Search terms were combined and used to search the following online databases: BNI, CINAHL, Embase, Medline, PsycINFO, Cochrane Registers, Web of Science and Google Scholar. Each database was searched from its inception through to November 2010, with no language restrictions. In addition, hand searches of the following psychiatric journals were carried out: *American Journal of Psychiatry, Annals of General Psychiatry, Archives of General Psychiatry, International Journal of Social Psychiatry, British Journal of Psychiatry, The Psychiatrist, and Schizophrenia Bulletin*. Due to the specificity of the research topic, grey literature was identified through electronic searches of SIGLE (System for Information on Grey Literature) and The British Library Catalogue. This prompted hand searches of charity reports, information packs, case reports and published undergraduate/PhD dissertations. References from bibliographies of identified articles were analysed and relevant citations were selected for review. Frequently cited authors were contacted for expert information and literature recommendations.

Titles and abstracts were inspected to identify relevant reviews. A second independent researcher was allocated a random selection (20%) of abstracts for screening to determine inclusion. After agreements on ambiguous texts were reached, full texts of potentially relevant papers were obtained. Texts were retained if they met the following criteria: (i) participants were unpaid lay/nonprofessional volunteers; (ii) the volunteer activity was a regular commitment (e.g. not a ‘one-off’) with an adult mental health population^a^; (iii) the volunteering activity involved face-to-face contact and provided direct care. Texts were excluded if: (i) volunteers were family members, paid carers, paid lay workers, mental health professionals or already known friends; (ii) the volunteering activity was not specific to a mental health population (e.g. HIV/AIDS/asylum seeker/general hospital volunteering); (iii) the volunteering involved no direct face-to-face care (e.g. telephone helpline/online volunteering); (iv) volunteering was part of a course requirement; (v) the volunteering was a one-off activity (e.g. helping after a natural disaster); (vi) or the literature was inappropriate extraction material (e.g. a review paper or charity advertising booklet). Identified final texts were examined independently by two reviewers (CH, SP) to confirm inclusion.

### Data collection and extraction

Data were extracted independently by two reviewers (CH, GK), with a third reviewer adjudicating in the event of disagreement (CL). The extraction instrument allowed both qualitative and quantitative documentation of the study, including; study details (author, title, year, country, study setting, aims, methods, recruitment to study); volunteer socio-demographics (number, age, gender, education level, employment status, religion, ethnicity, relationship status, living arrangements); and volunteer characteristics (motivations; previous experience in mental health volunteering; previous connection to organisation; previous service user; volunteer role; volunteer activities; length of commitment; positive and negative experiences). Additional information was collected about the volunteer organisation (type of organisation; philosophy/aims of organisation; client group supported; benefits to persons with a mental illness; method of volunteer recruitment; volunteer selection criteria; matching process; volunteer training/support provision). Direct quotes from volunteers and persons with a mental illness were also extracted from papers, and these were reproduced in our results section to illustrate our findings.

## Results

**Figure 1:**
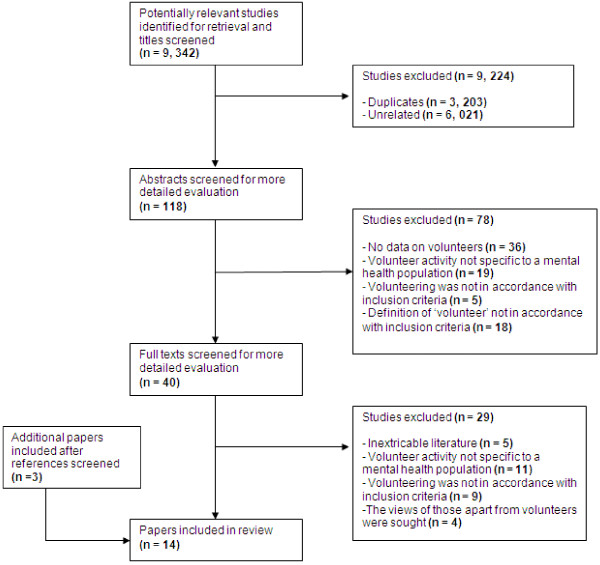
QUORUM flow diagram for paper selection.

Figure 1 shows a QUORUM diagram with the results of the literature search and the selection of papers. In total, 14 papers were included in the review.

### Overview of papers

Papers were published between 1967 and 2011. Six studies came from the UK, four from Germany, three from the USA, and one from Switzerland. All identified papers were written in either English or German. Eight were naturalistic evaluations, descriptions or reviews of a single volunteer programme, four were large population surveys but still obtaining data on volunteering, and two were small questionnaire studies. Of the ten papers that interviewed volunteers, eight interviewed less than 30 volunteers and two interviewed more than 100. In total, the review included data of 540 mental health volunteers.

### Volunteering programmes

Volunteers worked for programmes run by third sector, non-profit organisations, such as befriending or counselling schemes [21-27] or for programmes run by psychiatric hospitals [26,28]. The most frequently reported aim amongst these programmes was ‘patient social and community enhancement’ [22,24,27].

The information provided on the contexts in which the volunteers worked varied and was more detailed in papers that profiled a single service. These included befriending services attached to a psychiatric rehabilitation unit [25] and a community alcohol team in the UK [22]. Another befriending service was set up by parishioners from a local church with funding from local statutory authorities in Hastings, UK [24]. A local ‘intentional friendship programme’ was run by a non-profit organisation in a medium-sized northern city in the USA, with nearly 100 affiliate offices across the USA [29]. One volunteer reported of her time in a university linked psychiatric consultation service in Chicago, USA [28].

Most schemes asked for a minimum length of commitment from volunteers to enable a successful volunteer-client relationship. On average this was 12 months, but actual relationship length varied between volunteer-client pairs [24-27,29,30]. The highest level of commitment recorded was 5 hours a week [21], with the lowest at 4 hours a month [29]. Some organisations pre-matched the interests of the volunteers and people with a mental illness in order to increase the likelihood of a successful relationship. Factors such as gender, location, age and interests were typically taken into consideration [22,25,27].

Volunteer training and supervision were compulsory elements of most schemes [21-25,29], although some volunteers received no training [30,31]. Examples of topics covered in training sessions included: expectations and responsibilities of a volunteer, preparation for managing initial meetings, general listening skills, boundaries and guidelines, mental illness, stigma, major diagnoses and symptoms, and conflict management [22,29]. Supervision for volunteers was offered in the form of monthly multi-disciplinary meetings, one-to-one supervision sessions or telephone support [22,24-26,31].

Information on patients’ diagnoses was infrequently reported. Only five papers mentioned specific diagnoses, including: schizophrenia, manic depressive psychosis, depressive neurosis, anxiety states, dependent personality disorder, and alcohol addiction [22-26]. Others referred to the ‘chronically/severely mentally ill’ [29,31-33], ‘psychiatric patients’ [28,30], and ‘general mental health population’ [21,34].

Three papers described the means by which volunteers were recruited [22,24,25]. The most common method was adverts in local newspapers. Additional methods included: poster displays, word of mouth, local radio adverts, ceefax, handbills, and undergraduate/graduate enquiries.

Three schemes reported selection criteria for potential volunteers [22,25,26]. One befriending programme rated potential volunteers from 0 to 10 on the criteria: ‘reliable, responsible, conscientious, has initiative, adaptable, prepared to receive and accept feedback, good listening skills, non-judgemental, ability to learn new skills, and awareness of boundaries’ [22]. Only those who scored 6 or above in 7 out of the 9 items were invited to interview. One organisation required ‘intelligent, dedicated and motivated people’ [26], and another recruited only ‘current psychology undergraduates or graduates who have expressed a desire to do clinical psychology training’ [25].

One paper listed favourable volunteer characteristics from the vantage point of the service user and the mental health professional [31]. Persons with a psychiatric illness requested volunteers to be ‘a nice person, funny but not curious, intelligent, open to the world, good at thinking far ahead, finished studies, able to deal with conflicts, self assured, eloquent, active, and have some life experience’. Mental health professionals required volunteers to be ‘physically healthy and stable, no need of own psychiatric help, self reflective, to be able to take initiative, active, sensible and able to listen.’

### (i) Characteristics of volunteers

#### Age

Three papers provided both the average age and age ranges of their volunteers. These were 44 (25–64), 36.6 (23–48) and 50 (29–65) respectively [21,22,27]. Three gave age ranges only (18–59), (21–27), (16–76) [23,31,34], and three provided loose qualitative descriptions [25,29,30] (See Table 1 for details).

**Table 1:** Summary of papers included in the systematic review

**Country**	**Year**	**Study design**	**Number of volunteers interviewed**	**Volunteer age**	**Volunteer gender (%)**	**Type of volunteering**^**§**^	**Type of mental health population**
Germany [32]	1995	Large opinion survey of general public in Old and New Länder	9 of 1005 interviewed in Old Länder were volunteers. 13 of 2089 in New Länder were volunteers.N = 22	No detail	No detail	Mixed*	Chronically mentally ill
Germany [33]	1994	Large opinion survey of general public in New Länder only	13	No detail	No detail	Mixed*	Chronically mentally ill
Germany [30]	1996	Large opinion survey of volunteers of 452 services in one German region	330 out of 898 responded to the survey (Response rate 37%)	68 · 5% over 50. Only 10% under 40	M (17 · 3) F (82 · 7)	Mixed*	Psychiatric patients
Germany [31]	1990	Small questionnaire study	13	(21–27)	‘Mostly female’	Befriending	Chronically mentally ill
Switzerland [34]	2000	Large opinion survey of general public in Switzerland	106 of the 1737 interviewed were volunteers	(16–76) ‘Older people more likely to commit to volunteering’	M (38), F (62)	‘In a mental health setting’	General mental health context
UK [21]	2010	Naturalistic study, service evaluation	12	44 (25–64)	M (25), F (75)	Counselling	Mental health problems - outpatients
UK [22]	1998	Naturalistic study, review of service	6	36 · 6 (23–48)	M (33 · 3) F (66 · 6)	Befriending	Alcohol addicts - outpatients
UK [23]	1989	Naturalistic study, description of service	30	(18–59)	M (26 · 6) F (73 · 3)	Befriending	Isolated and lonely users of outpatient psychiatric services. Diagnoses: Schizophrenia, manic depressive psychosis, depressive neurosis, anxiety states, dependent personality disorder.
UK [24]	2003	Naturalistic study, profile of service	No detail	No detail	No detail	Befriending	Socially isolated outpatients experiencing long standing mental health problems. 36% Have depression, 10% dual diagnosis, 54% misc (schizophrenia, manic-depression, anxiety, isolation, and long term-mental health problems).
UK [25]	2003	Naturalistic study, profile of service	No detail	20s as all undergraduates/graduates	‘Problems recruiting enough male volunteers’	Befriending	People who are considered to have enduring or severe/complex mental health problems. 70% of the 450 known to the service have schizophrenia.
UK [27]	2011	Small questionnaire study	8	50 (29–65)	M (75) F (25)	Befriending	Adults (outpatients) who find it difficult to form and sustain friendships as a result of moderate to severe mental health problems.
USA [26]	1973	Description of the volunteer ‘Case Aid’ program	No detail	No detail	No detail	‘Case Aid’ volunteering	Mental health inpatients and outpatients. Most diagnosed with schizophrenia.
USA [29]	2009	Naturalistic study, service evaluation	12	Unclear. All but 1 participant estimated to be over 30, some of retirement age	M (33 · 3) F (66 · 6)	Befriending	People with severe mental illness (outpatients). Specific psychiatric diagnoses were not obtained
USA [28]	1967	Naturalistic study, single volunteer experience	1	No detail	F (100)	Member of a hospital psychiatric consultation team	Psychiatric inpatients

*Mixed = *A range of volunteering services are described in the paper.*

^§^Type of volunteering = *Rough equivalent of volunteer activity described by the paper. See individual papers for more detailed information.*

Total (N) = 540 (22 + 330 + 13 + 106 + 12 + 6 + 30 + 8 + 12 + 1).

#### Gender

Of the eight papers that reported gender [21-23,27-30,34], all but one reported a higher number of female volunteers [27]. One paper mentioned specific problems with ‘recruiting enough male befrienders’ [25], and another described their volunteers as ‘mostly female’ [31]. In one paper identifying willingness to volunteer, females were considered more likely to engage in volunteering [32].

### Employment status

Employment profiles were mentioned in four papers [22,27,30,31]. In the first paper, three volunteers were employed, one was unemployed, one was unwaged (mother in the home), and one was a student [22]. Of the eight volunteers in the second, four were retired, two were unemployed, one was studying, and one was engaged in other voluntary work [27]. In the third paper, of the 330 volunteers interviewed, 65% were not in full time employment and 16% were [30]. In the fourth, ten out of the thirteen volunteers were students, six of these students in psychology [31].

### Relationship status

Volunteer relationship status was included in three papers [22,30,31]. In the first paper, three were divorced, two were married and one was single [22]. Of the 330 volunteers in the second paper, 67% were married and 31% were living without a partner [30]. Ten out of thirteen volunteers in the third paper were living alone [31].

### Psychiatric history

One paper reported that those who had had their own experience of mental health problems were likely to engage in volunteer work [32]. This was illustrated in four subsequent papers by volunteers disclosing a personal psychiatric history; four out of twelve [29], three out of eight [27], two out of six [22], and 10.7% of 330 respectively [30]. Volunteers with their own psychiatric history acted as role models and inspirations to those with a current mental illness, as they were able to demonstrate that ‘life does go on’ and that ‘it is possible to cope with a severe mental illness’ [29].

### Previous volunteer experience

Two papers noted that their volunteers had had some previous experience of mental health volunteering [21,27]. In the first paper, three out of twelve volunteers had previous counselling experience [21], and in the other, three out of the eight had previous befriending experience [27].

### (ii) Reasons for volunteering

Reasons for volunteering were assessed in five papers, using different methodologies and resulting in a variety of responses [23-25,30,31]. Using one of the four dimensions (‘getting – giving’) from the ‘Octagon model of volunteer motivation’ [35] as a framework, we grouped volunteer motivations into broad categories of what they can ‘give’ to others and what they can ‘get’ for themselves.

#### ‘Giving’

At one end of the spectrum there are ‘giving’ motivational themes. These include: *philanthropy*: ‘desire to give something of themselves’, ‘desire to give something back’, ‘desire to help others’ [23,24,30]; and *social responsibility*: ‘It’s not [the patient’s] fault that they are in this situation – unlike, say criminals. We as a society will be partly to blame if we do not get involved in assisting them’ [31].

#### ‘Getting’

Motivational elements related to ‘getting’ included: *curiosity*: ‘to test out own suitability for a befriending role’, ‘to find explanations for own behaviour’ [24,31]; *personal needs*: ‘to acquire new skills’, ‘meet new people’, ‘to have close contact with others’, ‘to be accepted and liked’, ‘to enhance own awareness of mental health issues’, ‘to learn more about mental health services’, ‘to have a new social commitment after children have left home’ [24,30,31]; and *career development*: ‘to gain psychologically relevant experience’, ‘to test out career aspirations’, ‘because of a recommendation from contacts in the mental health/social work field’ [24,25,30,31].

### (iii) The experiences of volunteers

#### Positive experiences

Rössler and colleagues [30] reported that volunteers hold a very positive view of their work with people with a psychiatric illness. 87% indicated they were ‘rather or very’ satisfied with their work and had their expectations fulfilled, 75% ‘never or rarely’ thought about quitting, 46% experienced ‘no or nearly no’ conflicts during work, and 4% thought their work was *always* interesting and pleasant. 33% felt that they could do a better job than professionals.

Qualitative reports of volunteer experience were provided by two papers [27,29]. One positive outcome was the development of the volunteer-client relationship into ‘something more natural, much like a friendship’ [27]. For volunteers, gaining a new companion, with whom they could talk ‘openly and honestly with…in ways that they could not in other social and business circles’ was mentioned as a particular benefit for those involved in befriending relationships [29].

*‘When I’m talking to him I’m not constantly thinking of the roles that I’m the befriender and he is the befriendee; we’re two people having a chat.’*[27] [Volunteer].

*‘I like it that she’s been there even for me, when I needed someone to lean on, that I could talk to her.’*[29] [Volunteer].

Another positive experience reported was ‘feeling good about helping someone else’, mentioned by 8 out of 12 befrienders in one paper [29].

*‘No matter how much time, or lost sleep, or stress you feel the investment requires, the satisfaction of being intimately involved with another life in recovery is just extraordinarily self-enhancing, reinforcing.’*[29] [Volunteer].

*‘I feel good about myself that I’ve been able to do something for him.’*[29] [Volunteer].

Some volunteers reported positive experiences even when faced with personal challenges. Volunteering in mental health required individuals to deal with their own preconceptions about mental illness, and challenged their own social norms [27,29,31]. However, volunteers viewed these as ‘valued growth opportunities’ [29]. Individuals with no previous experience of mental health problems found volunteering an ‘eye opener’ to the difficulties and social stigma surrounding mental health [27]. Some reported that they ‘lost their initial concerns about [the unreliability of] people with a mental illness, and found them surprisingly normal’ [31].

*‘I don’t know anyone with a diagnosed mental disorder so I had no idea what someone like that would be like. Now it seems silly to sort of think about…It’s nice to sort of confirm that what you read in the papers isn’t representative of the mental health sector.*’ [27] [Volunteer].

Additional positive outcomes included: new perspectives of own mental health problems [27], feeling they were helped and had grown as much as the people with a psychiatric illness had [29], broadened horizons by doing activities that they ordinarily would not do [29], and increased self confidence [28].

Volunteers in one paper summarised that ‘the benefits of one-to-one volunteering far outweighed the cost in time, money and energy’ [29], with three volunteers having no negative comments about the process; ‘*They make it so easy for you - I don’t see any drawbacks’*[29].

### Negative experiences

Negative experiences were reported less often than positive experiences. One grievance amongst volunteers was that their role was often unclear. Some befrienders found themselves in more of a counselling or carer role, which did not always sit easily with being a friend [22,27]. Other volunteers found it difficult to assess the extent to which they were accepted and viewed as complimentary to paid professionals [22]. One hospital volunteer recalls feeling inadequate as ‘a layman among professionals’ [28].

Another source of negative experience was the volunteer-client relationship. 44% of the 330 volunteers in one paper experienced a ‘normal’ amount of conflict, whereas 4% experienced a ‘more than normal’ amount [30]. Concerns early on in the relationship were based on how to deal with resistances’ from people with a mental illness, whereas later concerns were focused on the ending of the relationship [27,28].

*‘I feel like it’s slightly kind of a bit like a taboo subject [ending the relationship]. Um, I think I would be scared of saying the wrong thing, if it came up.’*[27] [Volunteer]

Client behaviour was another factor in volunteer satisfaction. People with a mental illness who were ‘passive in decision making, inactive, inflexible or disengaged in their time together’, made volunteers feel unappreciated [29]. Those who used their volunteer ‘as a taxicab’ provoked ‘unpleasant feelings’ in the volunteer [29]. These feelings were further exacerbated when there were break downs in communication; clients failing to show up for scheduled activities, or being difficult to contact [29]. Volunteers also reported difficulties in knowing how to respond to information disclosed by the client. They found it difficult to balance being non-judgemental with their personal reaction [27].

*‘…the hardest thing is not giving a true reaction to the things she says, and biting my lip rather than making or voicing my judgements or opinions*…’ [27] [Volunteer].

### (iv) benefits for people with a mental illness

Three papers assessed the benefits for people with a mental illness in being involved with a volunteering programme [22,27,29]. The most consistently reported type of benefit was having a one-to-one friendship with someone outside of their immediate circle. Having a ‘casual, relaxed, informal interaction’ was of particular benefit to people whose most frequent exchanges were with ‘professionals with clinical agendas’ [29].

*‘It’s a great experience. I recommend it highly to people, especially people that have psychiatric problems. They need a friend, they need somebody to open up and talk to, and somebody they can be close to. You need it, a little intimacy, the friendship, the ability to talk to somebody other than your immediate [family*. [29] [Client].

Meeting someone who was already aware of their mental illness alleviated a lot of the initial anxiety people with a psychiatric illness often feel when making new friends.

*‘Some friends of mine in the system have said what do you need a befriender for, you’ve got a relationship, you’ve got friends. But actually this is more, somebody who’s aware of my history, it’s not like meeting a new friend whose first question is what do you do, why aren’t you working, what is wrong with you… it’s nice to dip your toe in the water by meeting someone, not as a friend, but meeting somebody fresh who knows your history but still respects you’*[27] [Client].

Clients also benefited from having a close companion who was intentional about pushing them outside of their comfort zone [27,29]. Volunteers encouraged clients to stand up for themselves in the face of families, employers, and the mental health system, and would introduced them to novel activities, or those that they were reluctant to do on their own [27,29]. As a result, clients would grow in ‘self-esteem, self-worth and self-confidence’, [22,29] and become more ‘outgoing, socially active, verbal, attentive to arrangements with others and flexible in accommodating others’ [29].

## Discussion

The review collated data on 540 volunteers reported in 14 papers. Only a few socio-demographic characteristics have been reported and we know little, for example, about the educational background and personal histories of volunteers. However, our results show that volunteers are an array of ages, a mix of genders (although slightly more females), have mixed employment status, marital status and a mix of previous own experience of mental illness. The majority of people are not in full time employment or are retired, which may help explain their ability to dedicate time to volunteering. The role of previous own mental health experience in influencing propensity to volunteer is unclear, but having a personal history does seem to be valued by some service users.

Motivations for volunteering can be grouped according to categories of ‘getting’ such as curiosity and ‘giving’ such as philanthropy and social responsibility. Overall, volunteers report positive experiences. People with a psychiatric illness benefit from having a volunteer by gaining a close companion from outside of their immediate circle, who does not stigmatize them, and helps to facilitate their social-community reintegration.

### Strengths

The review used a systematic approach to collate all published literature to date on the mental health volunteer population. It brought together a disparate literature, included papers in different languages (English & German), from across four different countries (Germany, Switzerland, England & USA) and highlighted that similarities exist across countries between mental health volunteers in terms of socio-demographics, motivations and experience.

### Limitations

Due to the disparate literature base, there was an increased risk of missing relevant papers using traditional search methods.

The review collated data on 540 volunteers which, despite being a substantial number, probably reflects only a tiny proportion of all volunteers in various programmes across the world. Indeed, only 14 relevant papers were found in our search, most of which provided poor information, with the bulk of the qualitative data provided by two papers [27,29]. In addition, there may exist a potential sampling bias in the methodology of some of the papers we reviewed. One paper reported that ‘participants in poorly functioning matches were not interviewed’ [29] suggesting that only volunteers reporting positive experiences were included. Volunteers with more negative experiences may have been purposefully excluded from other papers we reviewed or may have been unavailable for interview due to earlier drop out from the service. This would have implications for our conclusion that volunteers report overall positive experiences.

Another limitation was the lack of available information on patients’ diagnoses.

Finally, the information on the context of the volunteering schemes was scarce. The organisational context is likely to influence who volunteers. For instance, the befriending service in Hastings, (UK) ‘arose in response to an unmet mental health need within the local community’ recognised by local church parishioners in a town with high levels of deprivation [24]. Volunteers for this service were local Hastings residents who responded to an advertising campaign. In contrast, the befriending scheme based within the psychiatric rehabilitation service in Leicester (UK) was set up in response to ‘increased requests from undergraduates and graduates enquiring about shadowing or unpaid placements in order to gain experience for clinical training’, and subsequently all volunteers held an undergraduate psychology degree [25].

### Comparison with the literature

Few of our findings are unique to volunteers in mental health care. Volunteers have been shown to be a heterogeneous group in other organisations e.g. AIDS volunteers [36], and the motivations reported in this review are consistent with theoretical models of general volunteer motivation [35,37-39]. The most widely used model of volunteer motivation is the Volunteer Functions Inventory (VFI) [37] which identifies six functions relevant to volunteering: (1) developing and enhancing one’s career (career); (2) enhancing and enriching personal development (esteem); (3) conforming to the norms of, or establishing norms for, significant others (social); (4) escaping from negative feelings (protective); (5) learning new skills and practicing underutilized abilities (understanding); and (6) expressing values related to altruistic beliefs (value). Although we chose to categorise motivations according to the ‘getting’ and ‘giving’ dimension of the Octagon model [35], they could equally have been categorised according to the VFI criteria.

## Conclusions

The findings in this review have implications for the literature on lay attitudes towards people with severe mental illness. Whilst much of research has portrayed the public as those who hold negative stigmatizing beliefs, our findings present an alternative. We have identified members of the general public who regularly and voluntarily spend extensive periods of time with people with a psychiatric illness and report largely positive experiences in doing so. Future recruiters should target potential volunteers from a variety of backgrounds, as our collation of the literature suggests there is no ‘typical’ mental health volunteer.

We also found benefits of volunteer programmes for both clients and volunteers. Not only do people with a mental illness enjoy the novel companionship of volunteers, but they may also improve their social contacts and social inclusion as a result of continued volunteer support. Similarly, volunteers with little previous exposure to individuals with severe mental illness find themselves challenging their previous stigmatising assumptions. Such exposure for lay members of the public has potential implications for the reduction of stigma amongst the general public.

Given these possible benefits and the fact that volunteers are a relatively inexpensive resource, there is a need for specific research evidence on the best ways to implement volunteers in mental health services. There should be an interest in promoting volunteering and in designing programmes that are of specific benefit to both volunteers and people with a severe mental illness. For example, programmes would benefit from specific research on the best ways to recruit, train, support, and make use of volunteers within both inpatient and outpatient settings, without taking advantage of their freely provided time. Ultimately what is likely to be of most benefit is for future research to propose an integrative model of volunteering with clear theoretical and practical implications to persons with psychiatric illness, service providers, policy makers and other stakeholders in the field.

The findings may be particularly important in light of the funding cuts for mental health services that have occurred or are planned in many countries. Policies commonly emphasise that volunteers are no substitute to paid professionals. If this can be guaranteed, one may agree with the statement that ‘there is untapped (volunteering) potential within our communities that we cannot afford to ignore [20].’

## Endnotes

^a^The term ‘adult mental health population’ was used as an inclusive terminology and not a precise definition.

## Competing interests

The authors declare they have no competing interests.

## Authors’ contributions

CH, SP and GK contributed to the conception and design of the study. CH conducted the search, selected the studies, interpreted the data and drafted the manuscript. All authors were involved in the data extraction of the identified papers, contributed to and approved the final version of the manuscript.

## Authors’ information

At the time of submission, CH was affiliated with Queen Mary University of London. However as of 1^st^ October 2012 she will be affiliated with King’s College London. For future correspondence, please email: claudia.hallett@kings.ac.uk.

## Pre-publication history

The pre-publication history for this paper can be accessed here:

http://www.biomedcentral.com/1471-244X/12/226/prepub
